# The interferon regulatory factor 6 promotes cisplatin sensitivity in colorectal cancer

**DOI:** 10.1080/21655979.2022.2062103

**Published:** 2022-04-20

**Authors:** Lin Tan, Weiming Qu, Dajun Wu, Minji Liu, Qiongjia Ai, Hongsai Hu, Qian Wang, Weishun Chen, Hongbing Zhou

**Affiliations:** Department of Gastroenterology, The Affiliated Zhuzhou Hospital Xiangya Medical College CSU, Zhuzhou, Hunan, China

**Keywords:** IRF6, cisplatin, chemosensitivity, colorectal cancer

## Abstract

Colorectal cancer (CRC) is one of the most common malignancies and causes of cancer-related mortality worldwide. Cell proliferation and tumor metastasis as well as chemoresistance are correlated with poor survival of CRC. The interferon regulatory factor 6 (IRF6) is functioned as a tumor suppressor gene in several cancers and is associated with risk of CRC. We explored the role of IRF6 in CRC in the present study. The protein expressions of IRF6 in human CRC tissues, normal para-carcinoma tissue and liver metastases from CRC were measured. Cell proliferation, chemotherapeutic sensitivity, cell apoptosis, migration and invasion including the related markers along with IRF6 expression were explored. Our results indicated that IRF6 expression in CRC and liver metastasis were lower than normal tissues, which were correlated positively with E-cadherin and negatively with Ki67 expression in CRC tissue. IRF6 promoted CRC cell sensitivity to cisplatin to suppress cell proliferation, migration and invasion as well as aggravate cell apoptosis. Our study suggested that IRF6 may enhance chemotherapeutic sensitivity of cisplatin mediated by affecting cell proliferation, migration and invasion along with apoptosis through regulating E-cadherin and Ki67, while the identified molecular mechanisms remain to be further explored.

## GRAPHICAL ABSTRACT

**Figure:**
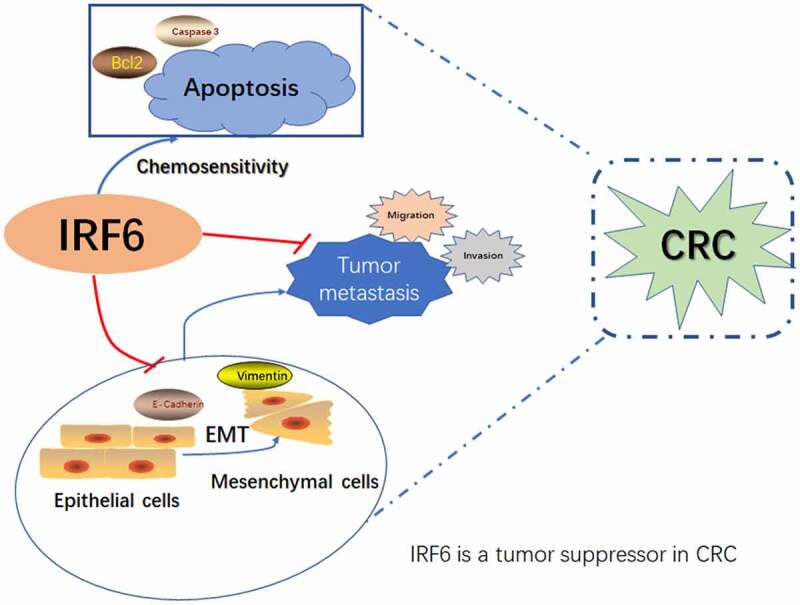

## Highlights

- IRF6 expression in CRC and liver metastasis was lower than normal tissues.
- IRF6 expression was correlated positively with E-cadherin and negative with Ki67.
- IRF6 increases CRC cells sensitivity to cisplatin in CRC.

## Introduction

Colorectal cancer (CRC) is one of the most common malignancies of the digestive tract, which is the third most common cancer in the world [1]. In recent years, CRC accounts for 10% of cancer-related mortality worldwide and becomes the fourth most common cause of cancer-related mortality [2]. Surgery is the mainstay curative treatment for non-metastasized CRC patients. The alterations in disease incidence, early screening as well as therapeutic improvements attributed to CRC-related mortality. The cure rate and long-term survival still need to be improved despite of surgical and medical therapies development in the past several decades, as cure rates and long-term survival have changed little. New treatments as neoadjuvant and palliative chemotherapy for primary and metastatic CRC have been developed. Chemotherapy remains one of the treatments for primary CRC after surgery and metastatic CRC. The sensitivity to chemotherapy is very important to the survival of CRC patients.

The interferon regulatory factor (IRF) family of genes directs the expression and activity of interferon and thus can strictly regulate innate immunity. The mammalian IRF family consists of nine members (IRF1-9). IRF recognizes common DNA sequences called interferon-stimulated response elements (ISREs) and functions as homodimer or heterodimer, interacting with other general mechanisms that regulates the transcription of transcription factors or genes [3]. Toll-like receptors (TLRs) represent a class of pattern recognition receptors, which can recognize certain molecular patterns associated with pathogens along with invasion, and are involved in a variety of diseases including cancer. TLR, on the other hand, triggers multiple signaling pathways of NF-κB, IRF and mitogen-activated protein kinase (MAPK) to produce various cytokines to associate with cancer and other diseases. Members of the IRF family have been reported to play a role as tumor suppressor genes in liver cancer, stomach cancer, colorectal cancer, breast cancer, prostate cancer and lung cancer [4–7]. IRF-5, IRF-1 and IRF-7 are associated with metastasis and prognosis of colorectal cancer [8,9]. IRF-6 has a similar structure to IRF5, and its association with cancer has been rarely reported so far. In macrophages, the IRF5 can regulate the expression of TNF receptor-associated factor 3 (TRAF3) by inhibiting its degradation [10], and IRF6 can form complexes [11,12] to participate in the degradation of IRF5. As TRAF3 is a key regulator of B cell survival and is considered to be a tumor suppressor gene of B lymphocytes [10,13], IRF6 was presumed to be a tumor suppressor. Studies have shown that IRF6 is low expressed in gastric cancer [14], and it has been recently reported that increased IRF6 expression can inhibit the proliferation, invasion and tumorigenicity of breast cancer cells and enhance the sensitivity of tumor cells to chemotherapy drugs [15]. The role of IRF6 in colorectal cancer has not been reported as far as we know.

Results of our preliminary experiments indicated that the expression of IRF6 in colorectal cancer tissues was significantly lower than that in paracancerous tissues, which was positively correlated with the survival of patients with CRC. It was presumed that IRF6 may be a tumor suppressor gene in CRC. We aimed to investigate the role of IRF6 in CRC in clinical samples detection and *in vitro* in the present study. Therefore, we further performed clinical samples assay, cisplatin sensitivity examination and cell function assay. The results of this study will provide data support for CRC treatment through exploring potential therapeutic targets.

## Materials and methods

### Materials

Cisplatin (PHR1624) was supplied by Sigma. IRF6 overexpression (IRF6-OE) and negative control (NC) were supplied by Shanghai Yuanke Biotechnology Co., Ltd. CCK8 cell proliferation and cytotoxicity assay kit (HY-K0301) were purchased from MCE. TUNEL kit (C1091) was purchased from Beyotime. lipofectamine 2000 (11668–019) was purchased from Invitrogen. Primary antibodies of Caspase-3 (19677-1-AP) and Vimentin (60330-1-Ig) were purchased from Proteintech. Antibodies of IRF6 (PA5-84583), BAX (MA5-32031) and Bcl2 (MA1-12246) were purchased from Invitrogen and E-cadherin (ab231303) as well as Ki67 (ab16667) from Abcam.

### Samples collection

All tissue samples including CRC tissues and adjacent normal tissues were collected from 2014 to 2015 and supplied by The Affiliated Zhuzhou Hospital to Xiangya Medical College CSU. CRC patients involved in the present study wrote informing consent under the approved protocol of our hospital. All clinical features were acquired from medical records of patients including gender, age, tumor size, TNM stage, degree of differentiation and regional lymph node metastasis. In total, 48 samples were obtained from patients without receiving any treatment as chemotherapy, radiotherapy or immunotherapy, nine samples from patients with chemotherapy prior to surgery and 52 samples received chemotherapy after surgery and followed up for 5 years.

### Hematoxylin-eosin (HE) staining

According to the previous study [16], the slices were dewaxed in xylene 2 times for 10 min each time, then in 100%, 90%, 80%, 70% alcohol successively 5 min each and dyed with hematoxylin for 5 min. After 5% acetic acid differentiation for 1 min, the slices were rinsed using water and then with acetic acid followed by being dyed with eosin for 1 min, dehydration in 70%, 80%, 90%, and 100% alcohol for 10 s each, then in xylene for 1 min. After the seal, photographs were taken under a microscope.

### Immunofluorescence

The expression of IRF6 was measured in samples using immunofluorescence based on the previous study [17]. The slices of samples were dewaxed by successively being put into xylene I (10 min), xylene butyl (10 min), xylene oxide (10 min), anhydrous alcohol fraction (5 min), anhydrous alcohol fraction (5 min), 95% alcohol (5 min), 90% alcohol (5 min), 80% alcohol (5 min) and 70% alcohol (5 min), then soaked in distilled water for 2 min and then incubated with primary antibody of IRF6 (1:50) overnight at 4°C in the wet box. After being washed with Tris Buffered Saline with Tween-20 (TBST) buffer, the samples were incubated with secondary antibody at 37°C in a wet box for 90 min. DAPI reagent was used to stain nucleus. The samples were observed by fluorescence microscope and photographs were taken.

### Immunohistochemical staining

The slices of samples were dewaxed similar to immunofluorescence and incubated with primary antibody of IRF6(1:200), E-cadherin (1:500) and Ki67 (1:500) overnight at 4°C in the wet box. After being washed with TBST buffer, the slices were incubated with HRP labeled secondary antibody at 37°C in a wet box for 20 min. In turn, we add color developing agent: rinse the sections with phosphate buffer (PBS) 4 times, 3 min each time, discard the PBS solution, dry the sections with absorbent paper, drop the newly prepared DAB color developing solution on each section and observe the slices under the microscope. The positive signal is brownish-yellow or brownish-brown. The time should be controlled well, do not develop too deep color, rinse the sections with tap water when it is acceptable and stop the color developing. Re-dyeing: Harris Hematoxylin re-dyeing is done, generally 30 s to 1 min, after washing with 1% hydrochloric acid alcohol differentiation, and then washing with PBS to return to blue. Dehydration: after rinsing the slices in water, the slices are successively put into 70% alcohol, 80% alcohol, 90% alcohol, 95% alcohol, anhydrous ethanol I, anhydrous ethanol p-xylene and e-xylene in the medium for dehydration and transparency and placed in each reagent for 2 min, and finally the slices are air-dried in the fume cupboard. For sealing, the neutral gum drops next to the tissue and then cover the cover glass, first put flat on one side, and then gently put down the other side, so as to avoid bubbles, and the sealed section lies flat in the fume hood to dry. The dried sections observed under a microscope or images can be captured.

### Tunel staining

Cell apoptosis of tissues from CRC patients without chemotherapy or with chemotherapy prior to surgery was analyzed using Tunel staining method according to the instructions of the commercialized Tunel kit [18]. The slices of samples were dewaxed in xylene for 5–10 min. The slices were switched to fresh xylene and dewaxed for another 5–10 min and anhydrous ethanol for 5 min. After treating with 90% ethanol for 2 min, 70% ethanol for 2 min and distilled water for 2 min, the slices were dropped 20 μg/mL DNase-free protease K in 10 mM Tris-HCl pH 7.4–7.8 and incubated at 20–37°C for 15–30 min and then washed 3 times with PBS. Note: this step must cleanse proteinase K. Otherwise it will seriously interfere with subsequent labeling reactions. Slices were incubated with 3% hydrogen peroxide solution (3% H_2_O_2_ in PBS) prepared by PBS at room temperature for 20 min to inactivate endogenous peroxidase. Then, the slices were washed with PBS or Hank’s Balanced Salt Solution (HBSS) for 3 times. 50 μL biotin labeling solution was added to the sample and incubated at 37°C for 60 min in the darkn. The samples were washed with PBS once, added to 0.1–0.3 ml labeled reaction termination solution and incubated at room temperature for 10 min. After being washed using PBS 3 times, the samples were added to 50 μl Streptavidin-HRP working solution and incubated for 30 min at room temperature. Then, it was washed 3 times with PBS and added in 0.2–0.5 mL DAB color solution, and the samples were incubated at room temperature for 5–30 min and washed with PBS for 3 times. Cell apoptosis was observed using a microscope and photographed.

### Cell culture and treatment

Human CRC HCT116, COLO205, SW-620 and SW480 cell lines were provided by the Cell Bank of Chinese Academy of Sciences (Shanghai, China). CCD-18Co (CRL-1459™) was supplied by ATCC. COLO205 and SW-620 were cultured in the medium of RPMI 1640 (31800022, GIBCO), HCT116 in DMEM (11965–092, GIBCO), SW480 in L-15 (41300039, GIBCO), CCD-18Co in MEM (11095098, GIBCO) supplemented with 10% FBS (11875–093, GIBCO-Invitrogen) and 1% Penicillin-Streptomycin (15070–063, GIBCO-Invitrogen) and incubated with 5% CO_2_ at 37°C. SW480 cells were transfected with plasmids of IRF6-OE or NC and treated with/without 0–40 μg/ml cisplatin for 48 h.

### CCK8 assay

10^5^/well SW480 cells were plated in 96-well plates and transfected with plasmids of IRF6-OE or NC and treated with/without 0–40 μg/ml (final concentration) cisplatin for 48 h. Then, the medium was removed and the cells were added 100 μl fresh medium with 10 μl CCK8 to the culture at 37°C for 2 h, the absorbance of each well was acquired by the enzyme plate analyzer at 460 nm [19].

### Migration and invasion assay

Cell migration and invasion assay were performed using Transwell plates (Corning, USA) according to the products’ introduction [20]. The treated SW480 cells were washed with 3 ml PBS, digested and collected with 0.25% trypsin, respectively, centrifuged at 1000 rpm for 5 min to remove the supernatant, moistened and washed using PBS twice. The cells were resuspended in serum-free DMEM medium and counted to dilute cell concentration to 5 × 10^5^cell/ml. 800 μl DMEM medium with 10% FBS was put into the 24-well plate and 200 μl cell suspension in Transwell upper chamber, respectively, then cultured at 37°C in 5% CO_2_ incubator. After incubation for 48 h, the Transwell was removed and the chamber was carefully cleaned with PBS, and then the cells were fixed with 70% glacial ethanol solution for 1 h, stained with 0.5% crystal violet solution and placed at room temperature for 20 min followed by being cleaned with PBS. The unmigrated cells on one side of the upper chamber were wiped with a clean cotton ball. Then, the migrated cells were observed and the photos were taken under microscope.

For an invasion assay, 100 μL Matrigel with a final concentration of 1 mg/ mL (BD, USA) was added to the Transwell to coat the upper surface of the membrane and then solidified by incubation at 37°C for 4–5 h to serve as the extracellular matrix for cell invasion analyses. After Matrigel dried into gel, 200 μL cell suspension of each group was inoculated in the upper chamber of Transwell and cultured in an incubator of 5% CO_2_ at 37°C for 48 h. The following progress is as same as the migration assay.

### Gene expression analyses

The mRNA expression of IRF6 of cells was analyzed using quantitative PCR (qPCR) [21]. 1 ml Trizol reagent was added to the cells with/without treatment, then transferred to 1.5 ml EP tube without RNase and lysed for 10 min to isolated total RNA. qPCR assay was performed using the qPCR Detection Kit (FulenGen, Guangzhou, China) according to the instruction of the kit. The specific primer of IRF6(NM_006147) is as follows: Forward primer (5’-3’) AACTGAACCCCTGGAGATGG and Reverse primer (5’-3’) GGTCCCCATAGAAGAGTCGG. Cycling profile of PCR was denatured at 95°C for 10 min, then 40 cycles of annealed at 95°C for 15 s, and extended at 60°C for 60 s. The average cycle threshold (Ct) of assays in triplicate was used for further calculations. The relative expression of mRNA was calculated using the 2− ΔΔCt method and fold change relative to the control group (CCD-18Co).

### Western blotting

Protein concentration of cells in RIPA lysate (P0013B, Beyotime) was detected using BCA protein concentration assay kit (P0010, Beyotime) according to the manufacturer’s instructions. The supernatant of the extracted protein was mixed with 5× protein loading buffer (P0015, Beyotime) (4:1) and placed in boiling water for 10 min. The proteins were separated by 12% SDS-polyacrylamide gels and transferred to PVDF membranes (Millipore, USA). The PVDF membrane was soaked in TBST containing 5% skim milk powder and sealed in a shaker at room temperature for 2 h. Then, the membranes were washed and incubated with the corresponding primary antibodies diluted with a blocking solution, and the PVDF membrane was immersed in the primary antibody incubation solution and incubated overnight at 4°C. The PVDF membrane was washed thoroughly with TBST for 5–6 times, 5 min/time. After that, the membranes were incubated with the corresponding horseradish peroxidase (HRP)-conjugated secondary antibodies and then were visualized by an enhanced chemiluminescence (ECL) system (Pierce Biotechnology, Rockford, IL). Anti-GAPDH (1:1000) and anti-IRF6 (1:1000) were used as primary antibodies. GAPDH served to normalize the results to correct for loading.

### Flow cytometric analysis

The cells were digested with 0.25% trypsin without EDTA, collected after the termination of digestion, centrifuged at 1500 rpm for 5 min and resuspended in PBS. Cell apoptosis was detected using Annexin V-APC/7-AAD cell apoptosis detection kit [22]. Cells were added with 5 μl 7-AAD dye solution in the 50 μl binding buffer and mixed, then 7-AAD dye was added into the collected cells and mixed, reacted at room temperature and kept in dark for 5–15 min. After reacted and mixed, 450 μl binding buffer was added. After that, 1 μl Annexin V-APC was added and mixed, then reacted at room temperature, away from light, for 5–15 min; (NC was also set, as normal cells were not added Annexin and 7-AAD; positive control 1, the solvent group with the most obvious apoptotic effect was used as positive control, only 5 μl AnnexinV single label was added; positive control 2, the solvent group with the most obvious apoptotic effect was taken as positive control, and only 5ul 7-AAD single label was added). Cell apoptosis was observed using flow cytometry (Beckman Coulter, cytoFLEX).

### Statistical analysis of data

All the data were analyzed using GraphPad Prism 8.0 software and demonstrated as mean ± SD from three independent experiments. Counting data were expressed as % and tested by χ^2^. Spearman rank correlation analysis was used for correlation analysis. The Kaplan–Meier method and log-rank test were used for survival analysis. The significant differences between groups were analyzed using the Student’s t-test. p < 0.05 is considered statistically significant.

## Results

### The expression of IRF6 in CRC tissues correlated with chemosensitivity and E-cadherin and Ki67

E-cadherin is one of the markers of epithelial-mesenchymal transition (EMT) which is related to tumor metastasis [23] and Ki67 indicated cell proliferation. To explore the role of IRF6 in CRC, the expression of IRF6, E-cadherin and Ki67 was measured in CRC tissue. The correlation of IRF6 expression with E-cadherin, Ki67 and chemosensitivity was also analyzed.

**Figure 1.**
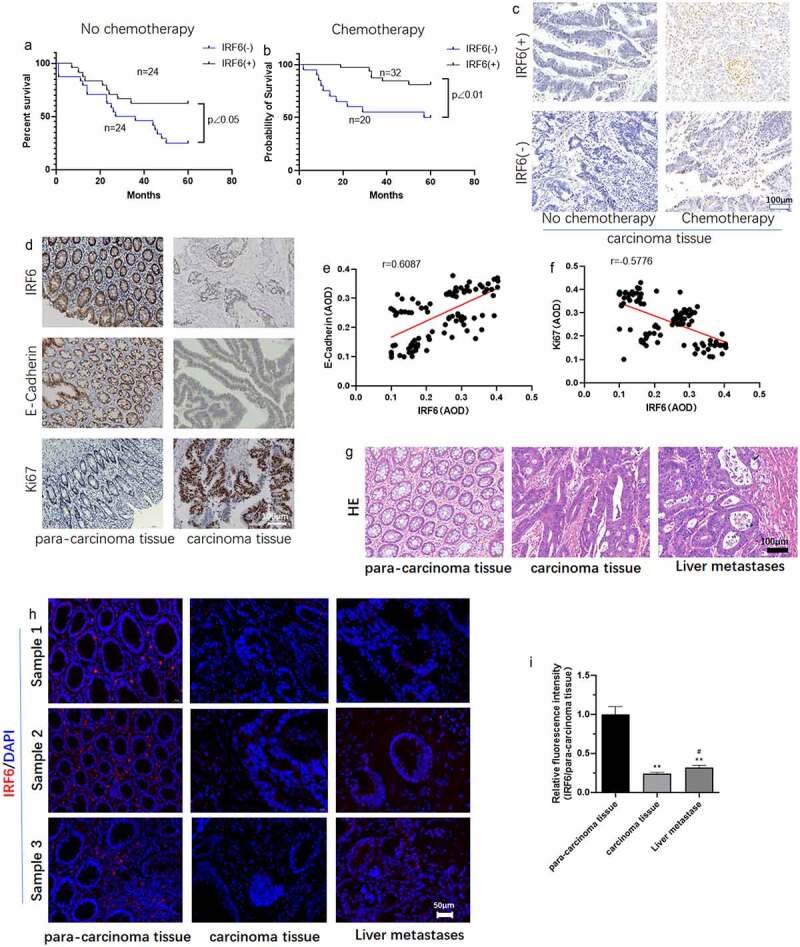
**The expression of IRF6, E-cadherin and Ki67 in CRC tissues and relation of IRF6 and survival of CRC patients, E-cadherin as well as Ki67 expression**. (a) Survival curves of CRC patients with IRF6 low expression or no expression (IRF6(-)) and high IRF6 expression (IRF6(+)) as well as without chemotherapy. (b) Survival curves of CRC patients with IRF6 low expression or no expression (IRF6(-)) and high IRF6 expression (IRF6(+)) as well as with chemotherapy after surgery. (c) Tunel staining to analyze cell apoptosis in CRC tissues without chemotherapy or with chemotherapy prior to surgery. (d) Immunohistochemical staining to analyze IRF6, E-cadherin and Ki67 expression in para-carcinoma tissue and carcinoma tissues of CRC patients with chemotherapy after surgery. (e) Correlation analysis of E-cadherin and IRF6 indicated by average optical density (AOD) value in CRC tissues. n = 100. (f) Correlation analysis of Ki67 and IRF6 indicated by AOD value in CRC tissues. n = 100. (g) HE staining; (h) IRF6 expression and localization were assayed using immunofluorescence staining. (i) Relative quantitative analysis for IRF6 expression. ** p < 0.01 vs para-carcinoma tissue; # p < 0.05 vs carcinoma tissue. n = 5.

The CRC patients involved in our study were grouped as IRF6(-) (low expression or no expression) and IRF6(+) (high expression) as well as with/without chemotherapy. As shown in Figure 1(a,b), survival of CRC patients with higher expression of IRF6 was significantly long compared with IRF6(-) patients, especially among the CRC patients undergoing chemotherapy that the p value is less to 0.01 between IRF6(+) and IRF6(-) group with chemotherapy as well as the p value is less to 0.05 among CRC patients without chemotherapy. We then measured cell apoptosis in CRC tissues using Tunel staining, results of which showed that the expression of IRF6 was positively correlated with apoptosis rate in CRC tissues of patients with/without chemotherapy (Figure 1c). It was similar to survival analysis that cell apoptosis in CRC tissues from patients with chemotherapy was prominent.

The expression of Ki67 and E-cadherin was assayed in CRC tissues and the correlation of IRF6 with Ki67 or E-cadherin expression was analyzed. It can be observed that the expression of IRF6 and E-cadherin seemed to be higher in para-carcinoma tissue than that in carcinoma tissue (Figure 1d). Correlation analysis results showed that the expression of IRF6 in CRC tissues is positively correlated with E-cadherin (Figure 1e), while negatively with Ki67 expression (Figure 1f). HE staining results of CRC tissues and liver metastasis from CRC were showed in Figure 1g. IRF6 expression was lower in CRC tissues and liver metastasis from CRC than that in normal para-carcinoma tissue indicated by the results of immunofluorescence staining (Figure 1h-i).

### IRF6 increases CRC cell sensitivity to cisplatin

**Figure 2.**
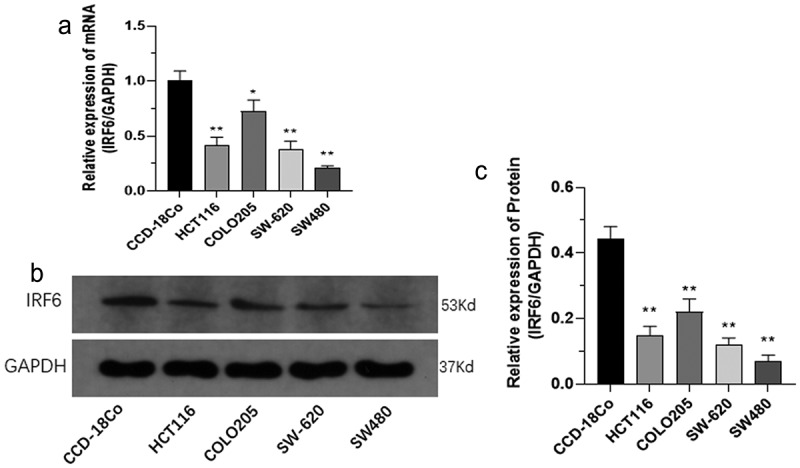
**The mRNA and protein expression of IRF6 was measured in CRC cells as HCT116, COLO205, SW-620 and SW480 cell lines along with CCD-18Co**. (a) The mRNA expression of IRF6 was measured in CRC cells as HCT116, COLO205, SW-620 and SW480 cell lines and CCD-18Co using qPCR. (b) The protein expression of IRF6 was measured in CRC cells as HCT116, COLO205, SW-620 and SW480 cell lines and CCD-18Co using western blotting. (c) The relative protein expression of IRF6 was analyzed using ImageJ denoted in gray. *p < 0.05, ** p < 0.01 vs CCD-18Co. n = 3.

To investigate the role of IRF6 in CRC *in vitro*, cytotoxicity test was performed in CRC cells transfected with IRF6 overexpression (IRF6-OE) plasmids. First, the mRNA and protein expression of IRF6 in four CRC cell lines and human normal colon tissue cells CCD-18Co were measured. Both the mRNA expression (Figure 2a) and protein expression (Figure 2(b,c)) were significantly low in CRC cell lines compared with CCD-18Co.

**Figure 3.**
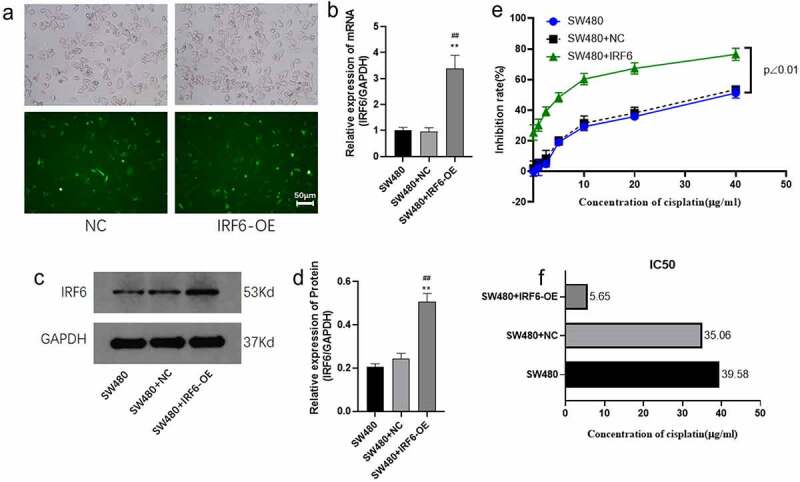
**Cell viability of SW480 transfected with IRF6 overexpression (IRF6-OE) or NC plasmids**. (a) SW480 was transfected with NC or IRF6-OE plasmids. (b) The mRNA expression of IRF6 in NC or IRF6-OE cells was measured using qPCR. (c) The protein expression of IRF6 in NC or IRF6-OE cells was measured using western blotting. (d) The relative protein expression of IRF6 was analyzed using ImageJ in gray. (e) Cell viability of NC or IRF6-OE cells was assayed using CCK8. (f) IC50 was analyzed using GraphPad Prism. ** p < 0.01 vs SW480. ## p < 0.01 vs SW480-NC. n = 3.

We then chose SW480 with the lowest IRF6 expression for further study. SW480 was transfected with IRF6-OE plasmids and the mRNA expressions along with protein expression of IRF6 were confirmed significant upregulated (Figure (3a–d)). Sensitivity of SW480 with/without transfected with IRF6-OE to cisplatin was explored in the followed study. As shown in Figure 3(e,f), cell viability was significantly decreased in IRF6-OE cells, and the IC50 decreased 5.21-fold in IRF6-OE cells compared with SW480 transfected with NC plasmids (SW480+ NC). According to the value of IC50, 6 µg/ml cisplatin was used in the followed study.

### IRF6 enhances the effect of cisplatin on CRC cell proliferation and apoptosis

**Figure 4.**
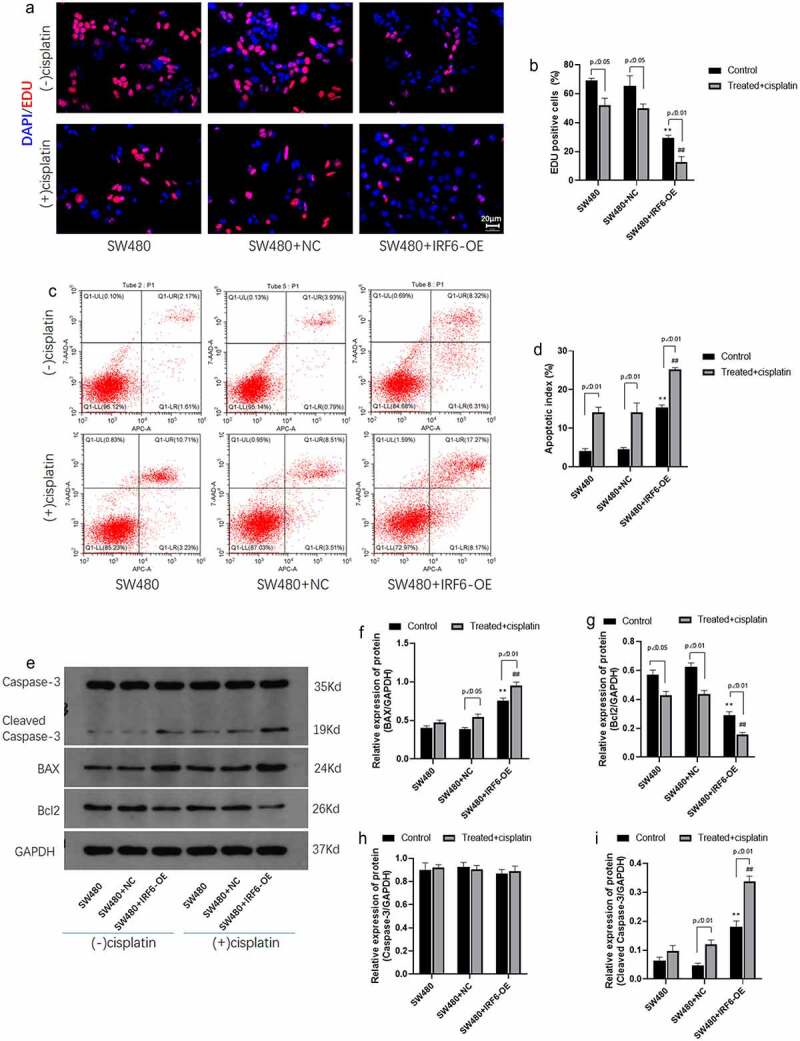
**Cell proliferation and apoptosis and related proteins expression in CRC cells were detected**. (a) Cell proliferation of SW480 transfected with NC or IRF6-OE plasmids was measured using EDU staining. (b) EDU-positive cells were analyzed using ImageJ. (c) Cell apoptosis was analyzed according to apoptosis assay by flow cytometer. (d) Cell apoptosis was measured by flow cytometer. (e) The protein expression of cell proliferation and apoptosis-related proteins, such as Bcl-2, BAX, Caspase-3 and cleaved caspase-3, was detected using western blotting. (f) The relative protein expression of BAX was analyzed by gray using ImageJ. (g) The relative protein expression of Bcl-2 was analyzed by gray using ImageJ. (h) The relative protein expression of Caspase-3 was analyzed using ImageJ in gray. (i) The relative protein expression of cleaved caspase-3 was analyzed using ImageJ in gray. ** p < 0.01 vs SW480-NC in control cells without treatment. ## p < 0.01 vs SW480-NC treated with cisplatin. n = 3.

The role of IRF6 in cell proliferation and apoptosis was further investigated in the present study. Results of EDU staining indicated that cisplatin inhibited cell proliferation mediated by EDU positive cells significantly and IRF6 enhanced cisplatin effect on cell proliferation (Figure 4(a,b)). Results of apoptosis assay by flow cytometer showed that apoptosis rate in IRF6-OE cells without treated with cisplatin were significantly increased as well as cisplatin-promoted cell apoptosis of control SW480 and IRF6-OE cells (Figure 4(c,d)). Cell proliferation and apoptosis-related proteins, such as BAX (Figure 4(e,f)), Bcl-2 (Figure 4(e,g)), Caspase-3 (Figure 4(e,h)) and cleaved caspase-3 (Figure 4(e,i)), were detected using Western blotting, results of which further confirmed that IRF6-OE promoted BAX and cleaved caspase-3 expression as well as inhibited Bcl-2 expression in SW480. Moreover, IRF6-OE enhances cisplatin-induced CRC cells apoptosis predominantly.

### IRF6 suppresses migration and invasion of CRC cells along with enhancement of the effect of cisplatin

**Figure 5.**
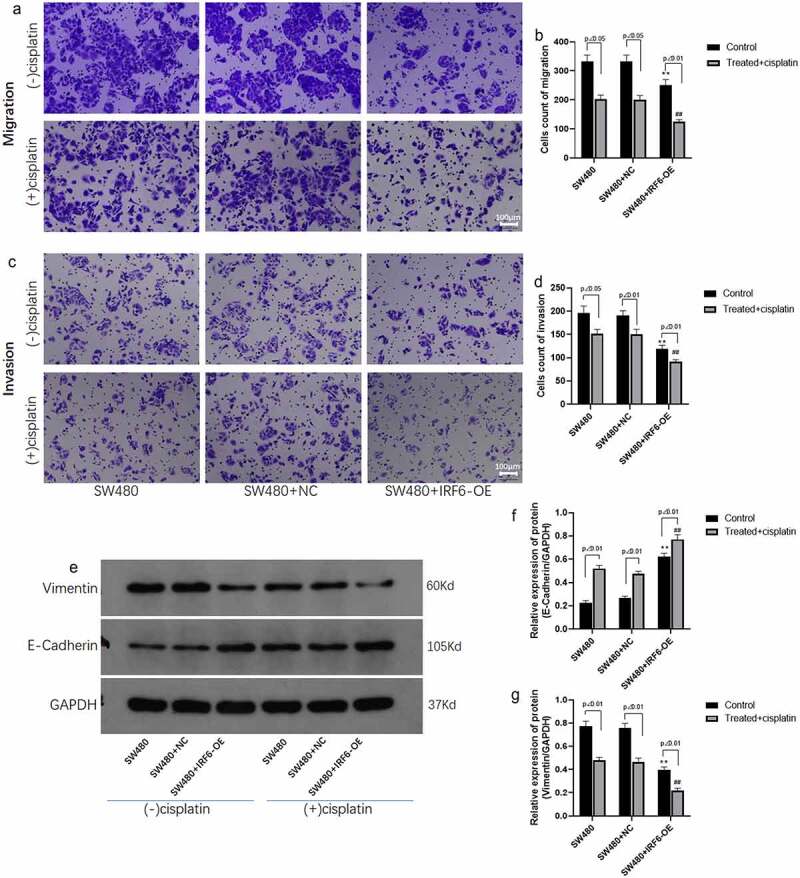
**Migration and invasion and EMT-related proteins expression in CRC cells were measured**. (a) Cell migration of SW480 transfected with NC or IRF6-OE plasmids was measured using Transwell. (b) Cell number of migrated cells was analyzed using ImageJ. (c) Cell invasion of SW480 transfected with NC or IRF6-OE plasmids was measured using Transwell. (d) Cell number of invasive cells was analyzed using ImageJ. (e) The protein expression of EMT-related proteins such as E-cadherin and Vimentin was detected using western blotting. (f) The relative protein expression of E-cadherin was analyzed by gray using ImageJ. (g) The relative protein expression of Vimentin was analyzed by gray using ImageJ. ** p < 0.01 vs SW480-NC in control cells without treatment. ## p < 0.01 vs SW480-NC treated with cisplatin. n = 3.

Tumor metastasis is related to the prognosis of the patient; therefore, the effect of IRF6 on CRC cells migration and invasion was explored using transwell method. It was shown in Figure 5(a,b) that the migrated ability of IRF6-OE cells was significantly decreased compared with NC cells undergoing cisplatin treatment or not. It was noted that IRF6 and cisplatin exhibited a synergistic effect on CRC cell migration. The invasive ability of CRC cells was investigated further, results of which were similar to migration assay that IRF6-OE suppressed cell invasion as well as promoted the effect of cisplatin on invasive ability of CRC cells (Figure 5(c,d)). As reported in the previous studies, EMT was associated with metastasis of tumor [24–26], and the EMT-related markers such as E-cadherin and Vimentin expression were detected in CRC cells of control and IRF6-OE (Figure 5(e–g)). Both in solvent control and cisplatin-treated cells, IRF6-OE upregulated protein expression of E-cadherin as well as suppressed Vimentin expression, especially in CRC cells treated with cisplatin.

## Discussion

CRC is one of the most common malignancies and the greatest contributors to cancer-related mortality worldwide [27,28], as metastatic CRC remains incurable in most cases. However, cytotoxic chemotherapy targeted to tumor and adjuvant chemotherapy for stage III patients will improve the survival of CRC patients [29]. Chemoresistance correlated with poor survival in CRC closely [30,31]. The sensitivity of tumor cells to chemotherapeutic drugs is very important for the prognosis of CRC patients. Rapid cell proliferation and metastasis of tumor contribute to survival of tumor cells and chemoresistance of CRC [32–35]. Previous studies have shown that numerous genetic markers associated with tumor cell proliferation and metastasis involved in molecular mechanisms of chemotherapy resistance, especially the genes identified responsible for CRC [36]. Bcl-2-associated athanogene 3 (BAG3) is related with tumor cell proliferation, migration, invasion and chemoresistance in colorectal cancer [37]. Production and accumulation of oncogenes as sphingosine 1-phosphate (S1P) often occurred in CRC [36]. S1P is known to regulate the processes to facilitate cancer cell proliferation, survival, migration, invasion and inflammation. S1P regulates CRC cell behavior and influences chemotherapy drugs treatment outcome [36]. Genomic profiling of CRC generates subsets of patients such as KRAS, NRAS or BRAF mutated and Her2 amplified, which will guide drug development and combination therapy approaches [38].

IRF6 is a transcription factor and is necessary for quiescence and differentiation [39]. IRF6 functions as a tumor suppressor gene in breast cancer [39], cervical cancer [40] and nasopharyngeal carcinoma [41]. In another study, IRF6 rs861020 was reported inversely associated with the risk of CRC [42]. In the present study, the IRF6 expression was detected in samples from CRC patients and the correlation of IRF6 expression with prognosis was analyzed. Our results indicated that the IRF6 expression was positively correlated with survival of CRC patients. It was worth noting that IRF6 expression enhanced the effect of chemotherapy on survival of patients. Ki67 is known to be associated with cell proliferation of cancer cells [43,44]. The expression of Ki67 in CRC tissues and the relationship of Ki67 with IRF6 was explored in the followed study, results of which showed that IRF6 is negatively with Ki67 in CRC tissues. IRF6 expression was found lower in CRC tissues and liver metastases from CRC compared with normal tissue adjacent to the carcinoma indicated by results of immunofluorescence staining. EMT involved in cancer progression as tumor cells migration and invasion are involved in tumor metastasis [45,46]. E-cadherin plays an important role in EMT [47,48]. We therefore investigated the E-cadherin expression in CRC tissues companied with IRF6. Our data showed that IRF6 along with E-cadherin was low expressed in CRC tissues compared with normal tissue adjacent to the carcinoma. It was also found that IRF6 was positively correlated with E-cadherin in CRC tissues.

We then conducted cell-level experiments to further explore the role of IRF6 in CRC progress and chemotherapy. Both mRNA and protein expressions of IRF6 were lower in CRC cells, such as HCT116, COLO205, SW-620, and SW480, than normal colon tissue cells as CCD-18Co. Although SW480 and SW620 cells are from the same patient, IRF6 expression is different in SW480 from SW620, this may be due to the fact that SW480 and SW620 are from different tissue and the IRF6 of expression in both SW480 and SW620 cells are lower than that in normal cells (CCD-18Co). The role of IRF6 in SW620 cells would be further explored in our future study. Results of cell proliferation and drug sensitivity assay indicated that IC50 of cisplatin in SW480 with overexpression of IRF6 was significantly decreased. IRF6 enhanced cisplatin effect on cells proliferation assayed by EDU staining. Cell apoptosis involved in IRF6 expression in CRC cells was further explored in our study, results of which showed that IRF6-OE strengthened the sensitivity of CRC cell to cisplatin observably which was similar to results of Tunel staining in CRC tissues with/without chemotherapy and confirmed by protein expression assay for cell proliferation and apoptosis-related markers as BAX, Bcl2 and caspase-3 (cleaved caspase-3).

Chemotherapy is one of the common intervention methods for metastatic CRC [28,49,50]. Tumor metastasis correlates with chemoresistance [51,52], which is involved in indicating a poor prognosis in CRC. The role of IRF6 in migration and invasion as well as EMT-related markers was investigated in the present study. IRF6 is conducive to inhibit migration and invasion of CRC cells. Moreover, cisplatin inhibited the migration and invasion of IRF6 overexpressed cells more significantly. And the results of EMT-related markers such as E-cadherin and Vimentin expression assay heightened the role of IRF6 in metastasis of CRC cells undergoing cisplatin treatment.

In our study, IRF6 expression was positively correlated with prognosis of CRC patients and inhibited cell proliferation and tumor metastasis but promotes chemotherapeutic sensitivity to cisplatin of CRC cells, which suggested that IRF6 may serve as a tumor suppressor in CRC, which was first reported to our knowledge.

## Conclusion

In conclusion, IRF6 is lower expressed in CRC tissue and cells than normal tissue or cells, which may be positively correlated with the prognosis of CRC patients mediated by suppressing cell proliferation and tumor metastasis as well as promoting cell apoptosis to enhance chemotherapeutic sensitivity of cisplatin. Although our results show that the expression of IRF6 was positively correlated with E-cadherin while negatively with Ki67 expression in CRC tissue, the identified molecular mechanisms involved in IRF6 and E-cadherin along with Ki67 remain to be explored in our further study.

## Data Availability

All data are available from the corresponding author if necessary.
